# Comparing the use of different environmental enrichment items by Hereford-Holstein cattle in a high containment facility

**DOI:** 10.1186/s12917-025-05211-y

**Published:** 2025-12-26

**Authors:** Rosanna Smith-Langridge, Rachel Jinks, Timm Konold, Anna Roberts

**Affiliations:** Animal and Plant Health Agency, Woodham Lane, Weybridge, KT153NB UK

**Keywords:** Hereford-Holstein cattle, High containment, Environmental enrichment

## Abstract

**Background:**

High containment facilities provide limited environmental stimuli for cattle. Adding environmental enrichment can reduce frustration and stereotyping, improving overall animal welfare and benefitting scientific output. Current research into environmental enrichment for indoor housed cattle is lacking despite such facilities facing unique challenges to maintain high welfare standards. This study compared four different environmental enrichment items in the aim to help inform high containment facilities on the most effective enrichment items for cattle. Five pens holding four 18-month-old Hereford-Holstein cattle were equipped with control enrichment (broom head and salt lick) and one trial enrichment item. The test items were a hay net filled with hay, rope, empty chemical drum, and ball. Items were rotated weekly over a three week period. Interactions between the cattle and enrichment were recorded daily via CCTV, data collected using continuous all-occurrence sampling with three point samples per day, and analysed using negative binomial regression models.

**Results:**

The hay net elicited the highest interactive frequency and duration. It was also the least affected by habituation, possibly due to the nutritional incentive and novelty created when refilled. A similar level of interaction was seen between the ball and drum and both items were interacted with more than control items. The rope was interacted with less frequently than control items.

**Conclusion:**

Although the hay net appeared most engaging, all items declined in popularity over time indicating that several different items rotated sporadically may maximise the benefit of enrichment by maintaining the cattle’s interest.

## Highlights

• Four enrichment items were tested on Hereford-Holstein cattle kept in a high containment facility over a weekly rotation period

• The hay net was interacted with most frequently and for the longest bouts of time

• All the novel enrichment items increased total interaction activity by the cattle

• Interaction with all enrichment items declined over time

## Background

Animal welfare is a very broad term that protects an animal’s wellbeing and is a crucial consideration when housing animals. It encompasses the maintenance of an animal’s physical health, provision of their needs, and their psychological state or feelings [1]. In the United Kingdom (UK) under the Animal Welfare Act (2006) it is the duty of whomever is responsible for the keeping of animals to provide them with a suitable environment in which they can exhibit normal behaviour patterns.. Animal welfare can be derived from their physical state, their behaviour and their ability to cope with their environment [2]. Behavioural measures, such as the frequency of play behaviour and stereotypies [3], as a proxy for freedom to express normal behaviour can therefore be measured to indicate a level of welfare. The preferences of the animal during choice tests can also provide invaluable information on what conditions are likely to improve their welfare [3]. Alternatively, there are physiological measures like veterinary health assessments [1], and heart rate [4]. However, these don’t necessarily consider the mental state of the animal and can be difficult to distinguish between excitement by negative or positive emotional states [4, 5]. Hagen and Broom [4] found that the heart rate of Holstein–Friesian heifers who had been given an operant conditioning task was increased compared with a control group that were subjected to the same conditions but without the task, indicating emotional stimulation from the activity. This may represent excitement or interest but could also be frustration. For the most accurate welfare assessment a holistic approach taking into account several differing indicators may be required [5, 6].

High containment animal housing facilities are used for animals in scientific research. “High containment” indicates that the research involves live pathogens which have the potential to cause serious harm to human and/or animal health (Advisory Committee on Dangerous Pathogens level 3 and Specified Animal Pathogens Order level 3 pathogens and higher [7]). Such facilities have strict biosecurity and biosafety requirements to prevent such pathogens from spreading to the outside world or from outside pathogens entering the study area. These requirements include preventing access from the study area to the outside (e.g. no windows or outdoor grazing), High Efficiency Particulate Air (HEPA) filtered air handling systems that use pressure gradients to prevent movement of air from infected areas to clean areas, specific protective equipment for all staff working in the area, and full chemical decontamination of the building between studies. These biosecurity measures result in the animals in these facilities being kept completely indoors, isolated from all other animals except those on their study, and restrict the amount and type of environmental stimuli that can be provided. The species and number of animals that can be housed depends on the requirements of the research and the size of the facility. The animals are separated into pens and have no interaction with those in other pens. The facility used in this study could house a maximum of 24 adult cattle, split into six pens of four cattle each. Permanent indoor housing for cattle is not only an aspect of high containment facilities,an increasing proportion of cattle farms are adopting year-round indoor practice [8, 9]. Cattle that live entirely indoors are faced with stressors and limitations that restrict their ability to carry out their natural behaviours [10], including reduced space for comfortable lying [11], abnormal social groupings, [9], and reduced time spent feeding [12]. Indoor housing can also reduce their physical health, such as the increased occurrence of lameness [8, 13], which provides a challenge for maintaining high standards of animal welfare.

A study using Holstein Friesian calves found that insufficient space inhibited performance of play behaviours and that calves released from a small pen (1.5m^2^ per calf) into a larger open arena (9.6 × 4.8 m) triggered the performance of significantly more play behaviour than calves released from larger pens [14]. Keeping cattle in an environment which provides space for both essential and non-essential activities, improves animal welfare and fitness [9]. For this reason, play behaviour and grooming are good positive indicators of cattle welfare [14, 15].

The 3Rs (Replacement, Reduction, Refinement) are a set of ethical principles required by UK legislation to be implemented by any institution conducting research on animals, throughout the process. Each is designed to target a different aspect of animal research and find ways to reduce animal suffering. Refinement is the principle that where possible a regulated procedure that an animal must undergo should be refined to reduce the pain, suffering and distress caused as much as possible [16]. This applies to the accommodation in which animals undergoing scientific procedures are held and the husbandry care they receive. Enriching their environment and providing the animals with species-specific behavioural outlets to improve their welfare is a key method of refinement [17].

Effective environmental enrichment can be described as an addition/modification to an animals’ surroundings which improves observed measures of welfare by providing stimuli which create opportunities for behavioural expression [18]. Environmental enrichment is designed to enable cattle to better cope with stressors in their environment by giving them increased available behavioural options [19], potentially mitigating stress and boredom [20, 21]. Enrichment can reduce frustration [22], agonistic behaviours [23], abnormal behaviours [23, 24], can increase fitness and physical health [9, 25], and positively affect weight gain [19, 26]. The provision of enrichment that provides an outlet for species-specific behaviours (e.g. grooming and oral manipulation for cattle) is mandated in the Animal Scientific Procedures Act (ASPA) 1986 UK code of practice and is essential to the welfare of indoor cattle; failure to do so may contribute to the development of stereotyping [27–29].

Habituation is a decrease in response to an external stimulus with long term exposure [30] and is a common occurrence with enrichment for many species including cattle [31]. For example Wilson et al. [32] found that Charolais-cross heifers began to lose interest in scent-releasing enrichment devices after the first day of exposure. The extent to which habituation to enrichment occurs depends on the item and the individual animal [23, 31]. When housed outdoors, cattle scratch and rub against objects such as trees to physically groom themselves [9, 33]. In indoor farming practices mounted brushes are a commonly utilised to enable the cattle to express this self-grooming behaviour [19, 34]. The presence of a brush in an indoor facility decreases the occurrence of stereotypic behaviours including headbutting, allogrooming, and bar licking [24] and may reduce frustration by allowing grooming of hard to reach body parts [35]. Adult cattle are highly motivated to access brushes [36], and never fully habituate to scratching enrichment suggesting it provides consistent long-term stimulation of a key behavioural need [23, 32].

It is unlikely for enrichment to be widely implemented unless it is considered by the people managing livestock to be practical for the facility, cost effective and with clear benefits to the animals [6]. In high containment facilities enrichment options are limited to what will not interfere with the research or husbandry work, and can comply with the waste management system (i.e. must be either disposable or able to withstand full chemical decontamination and must not impair the waste treatment process). With limited options but a high need for environmental additions, it is important to know which enrichment items are most effective. Different environmental enrichment will vary in the extent to which they benefit the cattle [6] and will provide opportunity for expression of different behaviours [37]. For physical enrichment to be considered successful, it should attract consistent attention from the cattle and allow expression of natural behavioural needs [25].

This study aimed to compare four different physical environmental enrichment items by analysing the duration and frequency of their use by twenty cattle. The preference of the cattle is assumed to reflect how beneficial the different environmental enrichment are to the cattle's welfare and thus their efficacy as enrichment [3]. The following items were chosen to encourage typical cattle play behaviours, which included oral manipulation, headbutting, chewing, pushing and scratching/rubbing [29, 38]. The chosen items were,a drawstring hay net filled with hay allowing for foraging and grooming, (separate to the ad libitum hay provided in their housing; an empty washed out 25L chemical drum that is anecdotally popular for headbutting behaviours; a rubber Kong ball with handles, allowing for oral manipulation and chewing behaviours as well as headbutting; and a large knot of natural fibre rope that could be used for chewing, licking and expressing oral behaviours. Items were rotated weekly over a three-week period.

## Materials and method

### Animals and housing

The study took place at the Animal Plant Health Agency (APHA) high containment research facility in England, UK. Cattle used for scientific research at APHA are protected by ASPA. This study was conducted in accordance with this legislation. Ethical approval for the concomitant research for which the cattle were being held at APHA was given by the Home Office under a specific project licence and by APHA’s Animal Welfare Ethical Review Board. Additional ethical approval for this study was not required as the provision of extra enrichment was above minimum standards and data were collected via Closed Circuit Television (CCTV).

**Fig. 1:**
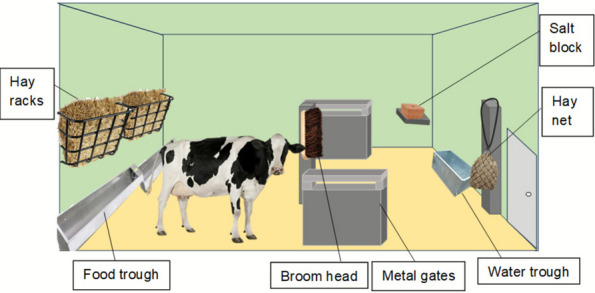
Pen layout. Diagram of the approximate layout of the animal pens with the broom head and salt lick in position. The hay net is placed as an example test enrichment item in the test location

Five identical pens (30.35 sq m) each containing four cattle (18 months old, male, Hereford-Holstein steers) were set up in a high containment facility. Pens were constructed of solid green painted walls with two solid access doors for staff on opposite sides (Fig. 1). Animals could therefore not see anything outside their pen. Pens had no natural light or furnishings, and were equipped with metal gates, metal feed troughs, metal automatic water trough, and metal hay racks filled with hay. Straw was used as bedding, which was changed daily.

All cattle were bought from an external supplier based in Denmark and were part of a concomitant scientific study into the immunogenicity of Bacillus Calmette-Guérin (BCG) vaccinations. The cattle had been reared in an open sided barn on a diet of concentrated feed and hay. They were given two weeks to acclimatise to the new facility before research was conducted. The concomitant study involved all cattle being vaccinated (half the cattle with BCG and half with a control substance) and then infected with *Mycobacterium bovis*, followed by regular blood sampling. It was assumed that neither vaccination status nor infection status would have any effect on their general behaviour since the vaccination was carried out several months earlier and Bovine tuberculosis is a chronic disease in which clinical signs are not seen at this stage. This concomitant BCG study dictated the sample size (four cattle per pen). No procedures or invasive practices were carried out for the duration of behavioural data collection, only routine husbandry. Routine husbandry involved one member of staff entering the pen at approximately 08:00 and moving the cattle to one side of the pen using the metal gates to create a barrier. All bedding from the empty side was shovelled into bins and replaced with fresh straw. Cattle were then moved to the clean side and the process was repeated. After this the cattle were fed concentrated feed (Cattle Rearer, manufactured by For Farmers) and the hay in the hay racks topped up. Staff members remained consistent throughout the study.

The cattle were fed 1 kg per individual of commercial concentrated feed twice a day between 08:00–08:30 in the mornings and 14:00–14:30 in the afternoons using communal troughs in each pen. Hay racks in the pens were filled with two bales of hay every morning resulting in ad libitum access to hay. The pens had an artificial light cycle that mimicked natural day lengths; lights came on gradually starting at 06:00 and turned off gradually at 20:00.

### Experimental design

The study ran over a three-week period with test items rotated each week. Each pen had a 60 cm broom head (made from wood with PVC bristles) and a 10 kg Equisalt salt lick (made from natural rocksalt without additives), providing an outlet for oral manipulation and essential nutritional salt supplement (Farm & Stable in UK). The broom head was tied to a central post with bristles facing out and the salt lick was placed on a salt lick holder mounted to the wall (Fig. 1). Brushes and saltlick were chosen as control enrichment for this study and were permanently placed in all pens. This prevented the cattle being without any effective enrichment for long periods of time and thus maintained an adequate standard of animal housing as laid out in the ASPA Code of Practice guidelines for cattle housing, which stipulates acceptable parameters in order to fulfil the requirements of sections 21 (5) of ASPA.

Test items consisted of a 106 cm hay net with large holes and drawstring (Farm & Stable, UK) filled with approximately 3 kg of hay during morning feed each day, a 30 cm red rubber kong ball with two large handles (Pets at home UK), an empty washed-out 25L plastic chemical drum (Brenntag, UK), and a large knot of natural fibre rope (Screwfix, UK). The test enrichment items were all hung at cattle head height from the same post that cattle had easy access to, in a position that ensured the items would not get in the way of the gates or husbandry work. One pen was always left with control enrichment only as a “control, no enrichment” on rotation. One test item was hung in each pen apart from the “control, no enrichment” pen. After a week the items were rotated between the five pens; this continued for a total of three weeks. Items were randomised between pens based on a partial latin square design such that no pen had the same item for more than one week and no pen was the “control, no enrichment” for more than one week (see Fig. 3). Items were not cleaned or replaced when rotated to a new pen so would potentially have residual traces of previous interactions. Cattle were observed via a CCTV camera placed in the top corner of each pen. The same person performed all the observations, avoiding intra-observer variation. Each pen was observed by continuous all-occurrences sampling for three five-minute intervals each day. Any interaction that began within that 5 min window was measured until its end, even if it continued for more than 5 min. Interaction frequency and duration with the enrichment and control items was counted and timed. An interaction was defined as a single animal making physical contact with the item. If two cattle were interacting with an item at the same time that was counted as two interactions. Interaction with the broom head and the salt lick were both counted as interaction with control items.

### Sampling frequency determination

**Fig. 2:**
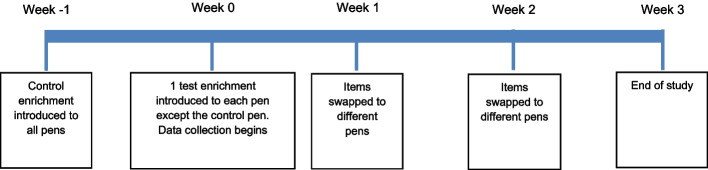
Study timeline. Timeline of the study showing enrichment item introduction and rotation

A short pre-study investigation was carried out to determine the most reliable sampling frequency to use to increase the validity of our methodology (see Fig. 2 for timeline). One pen of four cattle was given control enrichment items (broom head and salt lick), and cattle were observed via CCTV for five days. During this period, every interaction with the control items was timed and recorded. Using these data we determined that observation periods of five minutes at a time provided sufficient opportunity to observe interactions whilst remaining logistically feasible.

**Fig. 3:**
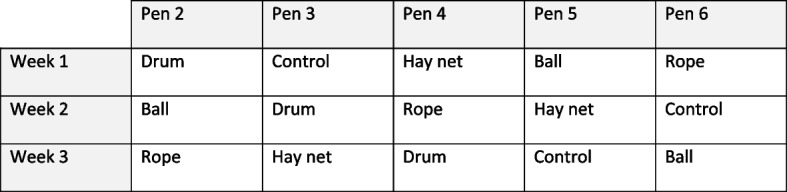
Enrichment rota. Experimental design table showing the placement of novel enrichment items across pens. “Control” indicates the absence of a novel enrichment item

Activity levels of the cattle were observed using instantaneous scan sampling every half hour during daylight hours (06:30–19:30) for one week. The number of active cattle were counted, with active being defined as “not lying down”. These data showed they were most active at feed times. However, this is not suitable for observing enrichment interactions as the entire period is spent eating and there is considerable interference from human presence. Therefore, the most active times at least one hour after feeding were used as the set observation points; these were 10:00 and 18:00. To maximise the representation gathered of the cattle’s behaviour across the whole day it was decided to also use a third observation point at a random time each day within daylight hours, which was determined using a random number generator. This is in accordance with Eisenhauer and Hanks [39] who found that irregular sampling using a combination of set and random time points increases the efficiency and accuracy of data collection in ethological studies (Fig. 3).

### Statistical analysis

#### Modelling interaction count data

A negative binomial regression model was used for the interaction count data, with the independent variables as follows:

- Novel enrichment type, factor variable. Factors: none (baseline), ball, drum, hay net, rope.
- Day of study, continuous. Values from 1 to 23.
- Observation slot, factor variable. Factors: 11am (baseline), 6 pm, the random time

Pen was included as a random effect. Interaction effects between enrichment type and day of study, and between enrichment type and observation slot, were also considered. The Akaike Information Criterion (AIC) was used to determine whether the addition of these terms improved the fit of the model to the data. However, it should be noted that the study was not originally powered to detect interaction effects and their addition caused problems with model fitting in some cases. Two different dependent variables were used in order to explore this data fully, firstly the total number of interactions with either the novel enrichment items or the control items (Model 1), and secondly the total number of interactions with only the control items (Model 2).

#### Modelling interaction time length

For these models only observation periods containing at least one interaction were included. The duration of the individual interactions within each observation period were summed and log10-transformed to produce an approximately normal distribution. A normal regression model was used to model this log-time data using the same three independent variables as described above and pen as a random effect. As described above, the interaction effects were assessed using the AIC. As for count data, the dependent variable is the total duration of interactions with all items (Model 3).

Data were entered in MS Excel and imported into Stata 15.0 [40] where the statistical analyses were performed.

## Results

Tables 1 and 2 display the raw data for the number of interaction and the length of interactions between the cattle and each novel enrichment item per day.

**Table 1:** Table of the raw data for the number of interactions with the test enrichment observed per day

Day	Drum	Hay net	Ball	Rope
1	7	4	4	1
2	0	0	0	1
3	1	0	2	0
4	1	4	0	0
5	0	0	1	0
6	3	1	1	1
7	0	0	1	0
8	0	1	2	1
9	2	1	4	1
10	1	3	2	0
11	2	1	0	0
12	0	3	0	0
13	0	1	0	0
14	2	0	0	0
15	2	1	2	0
16	0	1	0	0
17	1	1	4	0
18	0	1	1	0
19	0	2	1	0
20	0	1	1	0
21	0	0	0	0
22	0	2	0	1
23	1	1	0	0

Table of raw data of observed interactions the cattle had with the test enrichment items per day (totalling the three observation periods per day). Data are separated by enrichment item. The items were rotated into a different pen at the end of day 8 and 16 apart from the control enrichment which was present throughout

**Table 2:** Table of the raw data for time spent interacting with the test enrichment observed per day (seconds)

Day	Drum	Hay net	Ball	Rope
1	496	1620	1202	272
2	0	0	0	49
3	16	0	246	0
4	2	1111	0	0
5	0	0	9	0
6	123	49	80	3
7	0	0	42	0
8	0	190	418	6
9	57	217	248	178
10	31	1468	92	0
11	344	129	0	0
12	0	1377	0	0
13	0	315	0	0
14	767	0	0	0
15	737	106	238	0
16	0	202	0	0
17	44	33	96	0
18	0	401	13	0
19	0	115	323	0
20	0	215	235	0
21	0	0	0	0
22	0	124	0	49
23	39	80	0	0

Table of raw data for observed time the cattle spent interacting with the test enrichment items per day (totalling the three observation periods per day). Data are separated by item. The items were rotated into a different pen at the end of day 8 and 16 apart from the control enrichment which was present throughout

### Model 1: dependent variable is the count of interactions with all enrichments

Table 3 gives the results for Model 1. Adding any novel enrichment to the pens significantly increased the total number of interactions observed. Interaction levels dropped gradually over the time period of the study, and significant differences in total number of interactions were also seen between the three observation time slots. The cattle were significantly more active during the 6 pm slot and the randomly selected slot than the 11am slot.

**Table 3:** Results for Model 1: dependent variable is the count of interactions with all enrichments

Independent variable	Factor	Model coefficient	95% CI	*p*-value
Novel Enrichment	None (baseline)			
	Ball	4.29	2.11–8.75	< 0.001
	Drum	5.49	2.95–11.65	< 0.001
	Hay net	4.56	2.10–9.92	< 0.001
	Rope	2.85	1.36–5.99	0.006
Day	-	0.97	0.94–1.00	0.032
Observation slot	11am (baseline)			
	6 pm	2.43	1.55–3.83	< 0.001
	Random	1.81	1.13–2.90	0.013

A negative binomial regression model of the interaction count data, with independent variables of Novel enrichment type, Day of study, and Observation slot. Dependent variables were the total number of interactions with either the novel enrichment items or the control items. The baseline for the model is no novel enrichment at 11am on day 0. Coefficient < 1 indicates a negative relationship with interaction count; > 1 indicates a positive relationship

### Model 2: dependent variable is the count of interactions with control items only

The results from Model 2 are presented in Table 4. The addition of the drum and rope significantly increased the number of interactions with the control items, when compared to only the control items being present in the pen. Unlike Model 2 there is no evidence of a drop off in interaction with the control items over the period of this study as the Day effect is not significant. As in Model 2, the 6 pm and random slots have a significantly higher rate of interactions than the 11am slot.

**Table 4:** Results for Model 2: dependent variable is the count of interactions with control items only

Independent variable	Factor	Model coefficient	95% CI	p-value
Novel Enrichment	None (baseline)			
	Ball	2.15	1.01–4.60	0.048
	Drum	3.73	1.66–8.41	0.001
	Hay net	1.41	0.57–3.51	0.455
	Rope	2.44	1.13–5.28	0.024
Day	-	0.99	0.96–1.03	0.870
Observation slot	11am (baseline)			
	6 pm	3.52	1.96–6.35	< 0.001
	Random	2.41	1.31–4.45	0.001

A negative binomial regression model of the interaction count data, with independent variables of Novel enrichment type, Day of study, and Observation slot. Dependent variables were the total number of interactions with only the control items. The baseline for the model is no novel enrichment at 11am on day 0. Coefficient < 1 indicates a negative relationship with interaction count; > 1 indicates a positive relationship

### Model 3: normal regression: dependent variable = log (Total time interacting with any enrichment)

The results from Model 3 are presented in Table 5. All four novel enrichments increase the time spent with enrichment compared to only having the control items, but only the Ball and Hay net reach statistical significance. There is no significant effect seen for Day nor Observation slot on the duration of interactions (see Table 5).

**Table 5:** Results for Model 3: dependent variable is the duration of interactions with all enrichments

Independent variable	Factor	Model coefficient	95% CI	p-value
Novel Enrichment	None (baseline)			
	Ball	0.86	0.06–1.65	0.034
	Drum	0.22	−0.58–1.03	0.586
	Hay net	1.14	0.32–1.95	0.006
	Rope	0.36	−0.48–1.20	0.400
Day	-	0.011	−0.02–0.04	0.467
Observation slot	11am (baseline)			
	6 pm	0.26	−0.28–0.79	0.349
	Random	−0.08	−0.27–0.79	0.786

Linear regression model of the log-transformation duration of interaction (in minutes), with independent variables of Novel enrichment type, Day of study, and Observation slot. The dependent variable is the total duration of interactions with all items. The baseline for the model is no novel enrichment at 11am on day 0. Coefficient < 0 suggests a negative association between the factor and the dependent variable; > 0 suggests a positive association

For all three models, adding the interaction effect between enrichment type and day of study, and between enrichment type and observation slot, either resulted in a model with similar or higher AIC, or in a model which failed to converge. As such, interaction terms were not included in any of the models.

## Discussion

This study suggests that adult cattle will utilise additional and novel enrichment when given the opportunity indicating that it is advantageous for the cattle’s welfare to provide these resources [31]. This is demonstrated by the over 200% increase in total interactions with enrichment when a novel enrichment item was present compared with when only control items were present. All the enrichment items could be easily implemented in any high containment facility and did not interfere with research work. In an animal research facility good animal welfare is likely to have knock on benefits for the validity of data being collected. The physiology and condition of the cattle and other research animals can be significantly affected by their environmental conditions and behaviour [41, 4, 26]. High stress levels can impact the ability to obtain reliable data from animals so high standards of welfare are a necessity for valid scientific output particularly on long-term scientific studies [41, 42]. For example, it has been demonstrated that enrichment treatments led to lower lactate levels during handling for blood sampling in Gir x Holstein calves suggestive of lower stress levels [20]. Research procedures, such as collection of biological tissue samples from live animals are considerably safer for staff and animal when the cattle are less-stressed, since they are less likely to be reactive to handling [43]. This is a reason why refinements are of particular importance in a high containment research environment in which injury to staff could result in exposure to dangerous pathogens.

The broom head and salt lick were given as control items as these were deemed the minimum level of environmental enrichment that would maintain a sufficient standard of welfare for cattle in high containment as per the Code of Practices set out under ASPA 1986. The broom head allows for grooming behaviours and the salt lick for oral behaviours. Both these items are well established in cattle husbandry practices both on farms and in scientific housing [23, 24, 44]. Interest in the control items remained consistent throughout the study, indicating that they are not significantly affected by habituation (*p* = 0.870). The addition of novel enrichment to a pen did not cause any decrease in the use of the brush and salt lick, indicating that they do not compete with the test enrichment items. This confirms that they were good choices for control items and are a good minimum enrichment requirement for indoor cattle. They provide essential behavioural outlets, stimulating the cattle to perform species-specific behaviour.

With all the novel test items there was a significant (*P* = 0.032) decline in interaction as the study progressed and the novelty factor wore off. This was expected and is a well-documented aspect of cattle behaviour, although the extent to which habituation occurs has been shown to vary between individual cattle [30–32]. The initial peak in interest in the novel items can be explained by exploratory behaviour [30]. However, the novelty was short lived. This illustrates the value in regularly changing enrichment in pens and introducing novel items to maximise behavioural stimulation and mental interest [29]. A limitation of this study is that enrichment items were not cleaned before being introduced to a new pen. It is unclear whether the smell of other cattle on the enrichment items had any off-putting or encouraging effect. For future work, scent-based enrichment (such as used by [32]) could be added to these items in order to reintroduce novelty and increase interactions.

The time of day had an effect on the number of interactions. The cattle interacted with enrichment significantly more (*p* < 0.001) at 6 pm and the random time slot than the 11 am slot. Cattle are typically ruminating at 11:00; rumination in Holstein cattle peaked at 4 h after feeding [45], which for this study would be approximately 12:00. This demonstrates the importance of a fixed time schedule when assessing cattle behaviour.

The extent to which the enrichment items were interacted with varied between items. Out of the four items tested, the hay net was the most utilised. It was the item most frequently interacted with, the item they spent the longest time with and was used significantly more than control enrichment (*P* = 0.003). The presence of the hay net increased the total number of interactions with all enrichment in the pen more than fivefold and the time spent interacting by 4.5 times compared with control enrichment alone. It did not significantly increase the number or duration of interactions with the control items (*p* = 0.231), suggesting the total increase is due to the cattle interacting with the hay net. This matches the results of Zhang et al. [29] who found that singly housed Holstein calves with limited social contact showed a preference for interacting with a hay net over other items including a stationary brush and rope, despite having ad libitum access to hay in their housing.

The popularity of the hay net was most likely due to its greater number of interactive behaviour options and the daily novelty created by refilling it with hay each morning which may have better maintained the cattle’s interest. The hay net combines a play behaviour outlet incentive with a nutritional incentive and likely allows for the expression of normal grazing/feeding behaviour which is crucial for cattle welfare. Whilst interacting with the item, the cattle could alternate between eating and playing, such as headbutting and scratching [29]. Ruminants typically spend long periods of time engaged in feeding behaviours [46]. Cattle kept entirely indoors do not have access to grazing which affects their time budget. Play behaviour is typically an event of shorter duration, so to combine grazing/feeding with play could be a popular activity for indoor cattle and result in a large amount of interaction [35, 46].

The drum and hay net both had a great effect on the total number of interactions with enrichment, increasing the number of interactions by 6.303 and 5.538 times respectively (see Table 3). However, the presence of the drum significantly increased the number of interactions with the control enrichment (*p* < 0.001). This accounted for a large portion of the total increase and no statistically significant preference between the control items and the drum was found (*p* = 0.252). It is unclear why this effect was seen. It may be that the drum induced a state of excitement or curiosity which was then expressed using the control enrichments. Limitations of this study include the small sample size and short duration which make teasing apart the effects of the novel items from the effects of the control items more difficult.

The cattle showed a significant preference for the ball over control enrichment, and the presence of the ball increased the number of interactions with enrichment by a factor of 4.292 (*p* < 0.001, see Table 3). The ball is a squishy rubber texture with easily accessible handles making it good for chewing and other oral manipulation behaviours. It is also very mobile, being easily swung around and headbutted. This study observed that a popular form of interaction for the cattle was to swing the items around using their head, making full use of the large degree of motion available to them by the hanging installation. This is consistent with Strappini et al. [47] which found that weaned Holstein–Friesian calves showed a preference for a mechanical brush over a stationary one, suggesting they prefer the rotational, mobile tactile stimulation. Further research is recommended into the preference for mobile over fixed enrichment.

The knotted rope was of the least interest to the cattle. Although the presence of the rope did significantly increase the number of enrichment interactions observed (*p* = 0.006), it did so to a much lesser extent than the other novel items. Furthermore, the increase mostly consisted of increased interactions with the control items and there is no statistical evidence that the rope was preferred over the control items. The rope is the only item made from an absorbent material, therefore there would likely be stronger traces of the previous cattle and perhaps more damp from saliva on the rope compared to the other items. This may have affected its appeal. Alternatively, the rope may have been engaged with less than other items simply because of the reduced variety within the item. The other enrichments were hung from the post using the same rope and the cattle were occasionally observed chewing this rope while interacting with their item indicating that the behavioural outlet of chewing/scratching on rope can be gained within interacting with these other more varied items.

The data for the time spent interacting with enrichment support the conclusions from those of the number of interactions but less strongly (i.e. fewer statistically significant results). All the novel enrichments increased the total time spent interacting with enrichment compared with control enrichment alone but this was only significant for the ball (*p* = 0.028) and hay net (*p* = 0.003). The effects of time of day and day of the study were not significant for the time spent interacting with enrichment, whereas they were for the number of interactions (*p* < 0.001 and *p* = 0.032 respectively). There was only a weak relationship between number of interactions and time spent interacting, which may partly be due to the data collection methods used.

## Conclusion

This study reinforces the benefits of a grooming brush and salt lick as essential additions to cattle housing facilities to maintain sufficient animal welfare and found no habituation with these items. Of the novel environmental enrichment tested, the hay net was engaged with the most by the cattle, likely due to its multifunctionality. The provision of novel enrichment, regardless of item, increased the overall interaction activity which suggests the presence of novel items stimulates playful behaviour in the cattle. All of the novel enrichment items were subject to a decline in interest over time as the cattle became habituated, suggesting benefits to regularly rotating items. The effects of time of day on enrichment interactions confirms variability in behaviour across the day and reinforces the importance of a fixed schedule when assessing behaviour.

## Data Availability

The datasets used and/or analysed during the current study are available from the corresponding author on reasonable request.
